# First Investigation of a Eustachian Tube Stent in Experimentally Induced Eustachian Tube Dysfunction

**DOI:** 10.3390/bioengineering11101015

**Published:** 2024-10-11

**Authors:** Katharina Schmitt, Malena Timm, Philipp Krüger, Niels Oppel, Alexandra Napp, Friederike Pohl, Robert Schuon, Lisa Kötter, Marion Bankstahl, Thomas Lenarz, Tobias Stein, Gerrit Paasche

**Affiliations:** 1 Department of Otorhinolaryngology, Hannover Medical School, Carl-Neuberg-Str. 1, 30625 Hannover, Germany; schmitt.katharina@mh-hannover.de (K.S.); timm.malena@mh-hannover.de (M.T.); schuon.robert@mh-hannover.de (R.S.); koetter.lisa@mh-hannover.de (L.K.); lenarz.thomas@mh-hannover.de (T.L.); 2 bess pro GmbH, Gustav-Krone-Str. 7, 14167 Berlin, Germany; p.krueger@besspro.eu (P.K.); t.stein@bessgroup.com (T.S.); 3 Institute for Laboratory Animal Science and Central Animal Facility, Hannover Medical School, Carl-Neuberg-Str. 1, 30625 Hannover, Germany; bankstahl.marion@mh-hannover.de; 4 Institute of Pharmacology and Toxicology, Department of Biological Sciences and Pathobiology, University of Veterinary Medicine Vienna, 1210 Vienna, Austria; 5 Cluster of Excellence Hearing4all, Hannover Medical School, Carl-Neuberg-Str. 1, 30625 Hannover, Germany

**Keywords:** hyaluronic acid, stent function, nitinol stent, tapered stent, Eustachian tube, Eustachian tube dysfunction, ETD treatment, sheep, self-expandable metallic stent

## Abstract

Unmet needs in the treatment of chronic otitis media and Eustachian tube dysfunction (ETD) triggered the development of stents for the Eustachian tube (ET). In this study, for the first time, stents were placed in an artificially blocked ET to evaluate stent function. Eight adult female sheep were injected with stabilized hyaluronic acid (HA) on both sides to induce ETD. Subsequently, a tapered nitinol ET stent was inserted on one side, and animals were examined bilaterally by endoscopy, tympanometry, cone beam computed tomography, and final histology. Seven of the stents were placed in the desired cartilaginous portion of the ET. At the end of the study, one stented side appeared slightly open; all other ET orifices were closed. Tympanometry revealed re-ventilation of the middle ear in four out of seven correctly stented animals within 3 to 6 weeks after stent insertion. The major amount of HA was found at the pharyngeal orifice of the ET anterior to the stent. Thus, the stent position did not completely align with the HA position. While a functional analysis will require refinement of the experimental setup, this study provides first promising results for stent insertion in a sheep model of ETD.

## 1. Introduction

The Eustachian tube (ET) is a small tubular organ that connects the pharynx to the middle ear [1,2,3]. Its tissue composition of cartilage, bone, and ciliated mucosa [4] allows the physiological movement and, therefore, the function of the ET [2,5,6]. It mainly drains and ventilates the middle ear, preventing autophony and ascending pathogens. It therefore plays a very important role in the human well-being of patients. Eustachian tube dysfunction (ETD), the inability to maintain the ET functions [7,8,9], can be treated with non-invasive treatment options such as nasal irrigations (saline solutions) or nasal sprays (steroids). Surgical procedures described for ETD include laser Eustachian tuboplasty [10,11] and balloon Eustachian tuboplasty (BET) [12,13,14]. BET is now a well-established treatment [12] that has been described as safe and beneficial compared with tympanostomy tubes [15]. It is becoming more and more frequent in current surgical ETD treatment. Only very few severe complications are seen after BET, such as 0.3% occurrence of sensorineural hearing loss [16]. In addition, tympanograms show that BET can improve the middle ear pressure [17]. However, Hwang et al. [18] found that 39% of ears treated with BET did not show postoperative improvements in the tympanogram. Other study results showed that some patients did not benefit from BET, no matter how many times it was performed [19], or in a few cases, it even induced patulous ETs [20]. Therefore, clinicians need other treatment options for patients who do not respond to currently available ETD treatment options and/or need permanent support. Stents have come into focus as a possible solution for patients with acute and persistent ETD. In humans, ET stenting with rededicated cardiovascular stents and angiocatheters was successfully performed [21,22,23]. This highlights the need for stents developed for the special application in the ET. Stent prototypes have already been investigated in sheep [24] and pigs [25], and were tested in human cadavers [26,27], indicating that shape-adapted stents might be beneficial for the ET. More recently, shape-adapted designs were successfully tested in the ET in porcine models [25] and sheep [28]. All these tests were performed in healthy animals with naturally functioning ETs. So far, no implantation in ETs with proven ETD has been reported. However, an ETD model in sheep [29] was established. In the model, hyaluronic acid (HA) application (Restylane^®^ LYFT) induced ETD, which was confirmed by tympanometry. A randomized multicenter study [30] pointed out that the well-known stabilized HA filler Restylane^®^ LYFT is a safe injectable in plastic surgery. Restylane^®^ LYFT degrades gradually and is documented to mediate an aesthetic improvement for up to 12 months [30,31]. Similar approaches in ENT surgery are the treatment of a patulous ET with injections of hydroxyapatite [32] or HA (0.5to4.0 mL Restylane^®^ SubQ) [33] by the augmentation of the pharyngeal ET ostium. Based on these findings and the increasing call for ET stents, the current study was designed to test the application of a tapered nitinol stent in sheep with artificially induced ETD by HA injection. Sheep are close to the anatomy of the human ET [34] and are already proven to be a suitable animal model for ET stenting [24], tympanometry [35], and ETD [29].

## 2. Materials and Methods

### 2.1. Ethic Statement and Handling of Animals

The animal experiment was approved by the State Office for Consumer Protection and Food Safety of the Department of Animal Welfare of Lower Saxony, Germany, with reference number 19/3255 and complies with the Directive 2010/63/EU. The eight female German black-headed meat sheep (A1–A8) had an average age of 4 years (min.: 3 years; max.: 5 years) and were housed in the Central Animal Facility of the Hannover Medical School. The animals were acquired from a husbandry, where they were already familiar with contact to humans. They were stabled as a herd and had an average weight of 80.06 kg ± 12.74 kg (mean ± SD) on their day of arrival. Accommodation, usage, and care were in accordance with current animal welfare legislation for laboratory animals. The outdoor climate stables were lined with straw, and the animals had free access to tap water, hay, and pasture. The animal’s health status was regularly monitored by veterinarians throughout the study, and an established health score for sheep was used [29]. The score was applied every 2 to 3 days; after the endoscopic procedures, the animals were scored daily for 1 week. If health issues occurred, the scoring interval was also elevated to daily scoring. This procedure was used to ensure early detection of health problems of the animals, particularly changes in the upper respiratory tract, and to maintain the health and well-being of the animals. Vocalization, activity, feed/water intake, behavior/facial expression, breathing rate, and nasal discharge were examined.

### 2.2. Experimental Setup and General Anesthesia Protocol

#### 2.2.1. Experimental Setup

On arrival, all animals were first examined by a veterinarian and quarantined. Subsequently, the sheep were trained for 4 weeks to tolerate general handling with as little stress as possible and to obtain valid tympanometry measurements. To support the training and daily nutritional needs of the animals, complimentary feed pellets were fed (V5103-000, Complimentary feed for sheep and goats, 4 mm pellet, ssniff-Spezialdiäten GmbH, Soest, Germany).

A total of 5 endoscopic procedures were performed under general anesthesia (GA). An initial check-up including cleaning of the external auditory canal (EAC), bilateral augmentation of the ET for ETD induction, unilateral stent insertion, and two follow-ups at 1.5 and 3 months after stent insertion were performed in succession (see Table 1). The study had a total observation period of 3 months from stent placement. All animals followed the same experimental procedure, which was adapted from approved study protocols [24,28]. Cone beam computed tomography (CBCT), sample processing, and histology followed the in vivo part of the experiment.

#### 2.2.2. GA Protocol

The anesthesia protocol applies to every mentioned endoscopic procedure. Animals were deprived of food 18–24 h prior to the procedures but had free access to water at all times. They were initially sedated by intravenous (i.v.) application of 0.2 mg/kg i.v. Midazolam-ratiopharm 15 mg/3 mL (ratiopharm GmbH, Ulm, Germany), and received carprofen (2 mg/kg i.v., once following induction of GA; Carprosol^®^ 50 mg/mL, CP-Pharma, Burgdorf, Germany) for peri-operative analgesia. Anesthesia was then induced by i.v. administration of propofol (5–10 mg/kg i.v.; Narcofol^®^ 10 mg/mL, CP-Pharma GmbH). A gastric tube was inserted to prevent gassing of the stomach (rumen) or aspiration of gastric juice. Isoflurane (1.5–2.0% end-tidal concentration; Isoflurane CP 1 mL/mL, CP-Pharma) was administered via orotracheal intubation to maintain anesthesia, mixed with a 1:1 ratio of oxygen to air (average flow rate of 1 L/min).

The sheep were placed on the operating table in sternal recumbency in spontaneous respiration or artificially ventilated if necessary. Ringer’s solution (5–10 mL/kg/h, i.v., Ringer-Lactat-Lösung ad us. vet^®^; WDT, Garbsen, Germany) was administered by i.v. infusion throughout the procedure, and blood pressure, blood oxygen saturation, heart rate, respiration rate, and rectal temperature were monitored continuously. Following the GAs, the animals were returned to their stables and closely observed until they were able to walk and eat. Other animals from the study group were always present in the stables as a stress-reducing measure. This ensured that the herd animals were not alone before and after anesthesia. At the end of the last (5th) GA, the animals were euthanized with an i.v. overdose of pentobarbital (Release^®^, 300 mg/mL, WDT).

### 2.3. Endoscopic Procedures under GA

Endoscopy for all GAs was performed with a rigid endoscope (HOPKINS^®^ 70°, 4 mm diameter, 30 cm length, KARL STORZ SE & Co. KG, Tuttlingen, Germany) and allowed a full view of the ET orifice during the initial ear cleansing, augmentation, and stenting and at follow-up procedures. It was attached to a camera head (Telecam PAL, KARL STORZ), a fiber optic light cable, and to a Tele Pack Vet X LED RP 100 video system (KARL STORZ).

#### 2.3.1. EACs

Especially the first GA was used to clean the EAC from earwax. The solution Otodine^®^ (active substances: chlorhexidine digluconate, Tris-EDTA, pH 8.0; aniMedica (LIVISTO), Senden-Bösensell, Germany) was used to clean the EACs as needed, and ear forceps (221100 Hartmann EAR Forceps with very fine serrated jaw 1 × 4.5 mm, WK length 8 cm; KARL STORZ, Tuttlingen, Germany) were used to remove earwax if necessary under endoscopic guidance. From this point, Otodine was applied to each ear once a week 2–3 days prior to tympanometry measurements of the animals in the barn. In this way, the EACs were kept clean throughout the study. In addition, an assessment of the tympanic membranes (TMs) was conducted in every GA. The TM was evaluated as normal or, if abnormal, whether it was mildly scarred (mS), moderately scarred (S), distinctively scarred (dS), or bulging (B). Scarring in this context is used as an indicator of observed or suspected morphological peculiarities in the TM and does not reflect ETD symptoms.

#### 2.3.2. Pharyngeal Orifices of the ETs

During each GA, the pharyngeal orifices of the ETs were assessed. For this purpose, elongated swabs were inserted into both nasal cavities for 5 min beforehand. The swabs were soaked in one phial lidocaine hydrochloride 1 H_2_O (Xylocitin^®^-loc 2%, 5 mL, Mibe GmbH Arzneimittel, Brehna, Germany) for local anesthesia and xylometazolinhydrochlorid (Otriven^®^, 0.1%, 10 mL, 1 mg/mL, GlaxoSmithKline consumer healthcare GmbH & Co. KG, Munich, Germany) for decongestion. The pharyngeal ET opening was categorized for its degree of opening. A distinction was made between closed and open (slightly, distinctively, severely). Special findings were documented. In addition, the secretion character of ET discharge was evaluated for both ET sides as normal (clear, serous) or abnormal (opaque, mucous) (compare [28]).

#### 2.3.3. Injection of HA for ET Augmentation

The bilateral HA injection was conducted in the 2nd GA. The procedure was established and described in detail by Oppel et al. [29]. Briefly, non-animal stabilized hyaluronic acid (20 mg/mL HA, 3 mg/mL lidocaine hydrochloride; Restylane^®^ LYFT, GALDERMA, Lausanne, Switzerland) was used to augment both ET sides of each animal to artificially induce ETD. The HA was slowly injected into the mucosa under constant endoscopic control. The mucosal puncture was made in the lateral part of the ET just before the ET entrance. In this experiment, each animal could receive a volume of max. 5 mL HA to enable a sufficient blockage of the ETs’ function. The amount of injection was chosen individually during the operation. A veterinarian decided how much to inject. This was based on the visible mucosal protrusion and possible leakage of HA from the injection site.

#### 2.3.4. ET Stent Insertion

Stent insertion was carried out in the 3rd GA. The same insertion procedure, including the design of the insertion tools, was used as in the study of Schmitt et al. [28]. However, in this experiment, the stents were inserted into ETs that had previously been augmented with HA, and tympanometry (see Section 2.4) confirmed ETD. The choice of the side for stent insertion was based on successful ETD induction (see Section 3.2).

The nitinol stent (nickel–titanium shape memory alloy) was positioned onto the tip of an insertion tool [36] (bess medizintechnik GmbH, Berlin, Germany) and covered with a transparent outer sheath. The stent (length: 12 mm without; 14 mm with 3 X-ray markers at each end) reaches its maximum expansion at physiological body temperature. It tapers in the direction of the middle ear such that the shape of the ET is adapted (diameter: 5 mm decreasing to 3 mm).

The application tool (length: 30 cm) was inserted into the nasal cavity, the *Meatus nasi ventralis*, of the sheep. The endoscope was forwarded into the other nasal cavity. The metallic tool tip is bendable to adjust to different insertion angles (0°–45°), and ends in a sphere (approx. 2 mm). The tool handle was used to maneuver the tool tip with the stent up to the pharyngeal entrance of the ET. After the tool was slowly inserted into the lumen of the ET, the stent was released by withdrawing the outer sheath. Consequently, the stent deployed to its original shape. The stent insertion process ended with carefully retracting the tool out of the ET. A white depth marker visualized the insertion depth of the tool. It was evaluated if the marker was completely (100%), at least halfway (>50%), or less than halfway (<50%) inserted into the ET lumen. For correct placement, the surgeon aimed to reach a 100% insertion depth.

### 2.4. Tympanometry

Tympanometry should provide information on the ventilation status of the middle ear. After stent insertion, it provides information on whether the stent generates ventilation of the middle ear and thus reduces the ETD induced by the HA. Measurements began after the 1st GA. The middle ear pressure of both ears was examined according to published protocols [35] by using a Madsen OTOflex 100 tympanometer (GN Otometrics, Münster, Germany) calibrated to a customized adapter for sheep ears. Before the HA depot was injected, it ensured physiological ET function. The measurements were carried out weekly three times in a row for each side. The data were transferred to and analyzed with the help of the software OTOsuite^®^ (version 4.84, GN Otometrics). The tympanograms were evaluated following the established curve types for sheep tympanometry [35]: Tympanogram curves A/An were declared physiologic, while type B curves with low amplitudes or even flat curves were declared pathologic. Type B tympanograms could show ETD with possible otitis media with effusion, disruption of the tympanic membrane, or malfunctions during the measurement. The type C curves also indicate ETD; they are shaped like type A/An curves but have a shift of the amplitude maximum by more than 100 daPa into the negative range. The tympanometer was set to measure the pressure range from −600 to 200 daPa during all measurements. For evaluation, always the value that was measured at least twice within the three consecutive measurements per day was taken.

### 2.5. Imaging

**Figure 1:**
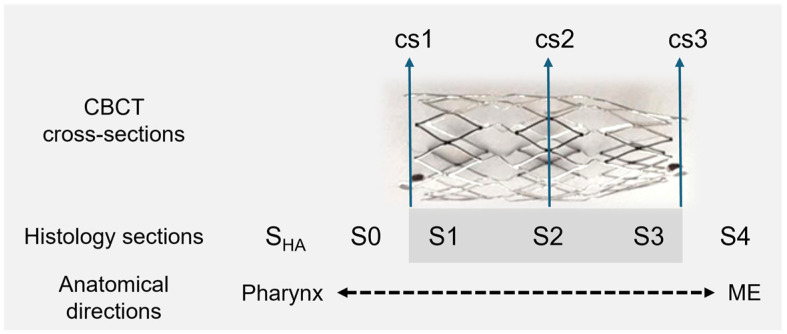
Schematic representation of CBCT measurements and histological sections. Nitinol stent, tapered from 5 mm (cs1, cs2) to 3 mm (cs3); ME—middle ear; S_HA_—section of HA; S0—section anterior to the stent; S1, S2, S3—stent sections; S4—section posterior to the stent.

Imaging of the heads followed directly after the 5th GA, which ended with the euthanasia of the sheep and the separation of the head from the body. The animals’ heads were put on ice and sent to CBCT. The CBCT scans (3D Accuitomo 170, J. Morita Mfg. Corp., Kyoto, Japan) were collected as an overview and detailed images for both sides (mode: CT, 360°; device settings: HiFi and HiRes imaging; 170 × 120 and 60 × 60, respectively). The software 3D Slicer [37] (https://www.slicer.org/; version 4.11.20210226; assessed on 17 March 2022) was used to analyze the DICOM files in terms of the position of the stent and its shape. The maximal and orthogonal cross sections (cs) were measured at both ends and the center of the stent (cs1—directed to the ET entrance/pharyngeal orifice; cs2—the middle; cs3—directed to the ET isthmus). The position of the cross sections matches the position of the histological sections S1, S2, and S3 (see Figure 1). The total length of the stent, including the radiographic markers, was also determined. The middle ear was assessed in terms of whether it was free, ventilated, partially filled, or completely filled with tissue and/or fluid.

### 2.6. Histology

Sample processing for histological examination began after imaging, on the day of the last GA. The ET samples and surrounding tissues were removed from the animals’ skulls (FK 23 bone saw, Bizerba, Balingen, Germany) and fixed in 3.5% formalin (pH 7.4; C. Roth, Karlsruhe, Germany) for 3 weeks. The formalin was changed twice a week. This was followed by a 10-day long dehydration process in a graded alcohol series (70%, 80%, 90%, 100%; ethanol; Merck, Darmstadt, Germany). The specimens were then infiltrated with methyl methacrylate (MMA) under vacuum. After 2 to 4 days in a water bath with up to 37 °C, the exothermic reaction was complete, and the samples were polymerized. Excess MMA was removed from the samples, and histological slices, ET cross sections, were produced with a saw microtome (Leica-SP1600^®^, Leica Biosystems Nussloch GmbH, Nussloch, Germany). Three consecutive slices were made for each defined cross section (S_HA_, S0, S1, S2, S3, S4; compare Figure 1) of the stent. Sections S_HA_ and S0 are located between the pharynx and the stent (anterior), and section S4 is located behind the stent in the ET lumen directing to the middle ear (posterior). After overnight drying (37 °C) in a metal press, the slices (thickness of approximately 45 µm) were stained with methylene blue (Löffler’s methylene blue solution; Merck) and Alizarin red (Alizarin red staining solution, Merck), and mounted on microscope slides using Entellan (Entellan^®^ Neu; Merck). The histological sections were examined with a digital microscope (BZ-9000^®^ and BZ-II-Analyzer program; KEYENCE, Osaka, Japan) at 2× and 4× magnification. For each section, multiple images were taken and merged.

Histological analysis was performed according to the study of Schmitt et al. [28]. The stent area (A_S_), the area of the lumen in the stent (L), the area filled by secretion in the stent lumen (S), and the area of granulation tissue in the stent (T) were measured. The remaining ET area and secretion outside the stent were defined as L_R_ and S_R_, allowing the total lumen (L_T_) of the ET (L_T_ = L + L_R_) to be determined. In addition to the existing method, the area occupied by the HA (A_HA_) was implemented and measured. For the control sides, the areas of the HA (A_HAC_), ET lumen (L_C_), and secretion (S_C_) were recorded. The cross sections of the control sides for L_C_ and S_C_ were prepared in analogy to the position of cross section S2, and the control sides for A_HAC_ were prepared in the same region as for A_HA_. Additionally, the stent area was divided into 4 quadrants to evaluate the epithelial lining of the ET lumen at different ET locations. The epithelium was graded as score 1 (>80%; intact), score 2 (>20–80%; intact but incomplete), score 3 (<20%; not intact), and score 4 (no epithelium). Score 4 indicated that the quadrant was fully ingrown with tissue. The same scores were also used for the control ET sides.

### 2.7. Statistics

Data were presented as mean ± standard deviation (SD). Statistical analyses were performed with GraphPad Prism 9 (GraphPad Software Inc., La Jolla, CA, USA). To detect statistical differences, a *p*-value of 0.05 was used. The data were checked for normal distribution using the D’Agostino–Pearson test (n = 8) and, if n < 8, the Shapiro–Wilk test. Afterwards, parametric or non-parametric statistical tests were applied according to the normality test results. An additional prerequisite for using a parametric test was normal distribution of the residuals. The paired *t*-test or Wilcoxon’s matched pairs signed rank test was used to evaluate the differences between the HA areas in histological cutting sections and the tissue in the stent area of the histological cutting sections. The Friedman test was used to analyze the epithelial scores of the quadrants. A mixed effects analysis was used to analyze the lumen of the different cutting sections, followed by a post hoc test (Tukey’s multiple comparisons test). Lastly, Pearson correlation analysis of the HA volume and the study week of the tympanometry data was conducted. Each presented individual value of a histological section represents the mean of three slices taken from this section. The stent sections of animal A7 were excluded from the statistical analysis due to incorrect stent placement (see Section 3.4).

## 3. Results

During the experiment, animals showed no signs of discomfort or were not conspicuous for diseases of the upper respiratory tract. The stent was well tolerated by the animals. However, one animal (sheep A4) had an elevated health score due to a lower respiratory tract disease. It was conspicuous with an elevated respiratory rate and elevated body temperature in study week 2 (6 days after stent insertion). Treatments were intramuscular injections with the antibiotic Hostamox^®^ LA (0.1 mL/kg body weight; 150 mg/mL Amoxicillin, MSD Tiergesundheit, München, Germany), i.v. injections of the antipyretic Vetalgin^®^ (20–50 mg/kg body weight; 500 mg/mL Metamizol-Natrium-Monohydrat, MSD Tiergesundheit), and Bisolvon^®^ (5 g oral; 10 mg/g Bromhexinhydrochlorid, Boehringer, Ingelheim, Germany) for secretolysis. The fever subsided after 1 day, and the treatment resulted in a physiological body temperature. Additionally, the sheep showed no elevated respiratory rate anymore and scored 0.

It should be mentioned that, during the 2nd GA, the method of intravascular ultrasonography (IVUS) was tested in three animals bilaterally as part of another series of experiments. In animals A5, A6, and A8, the IVUS probe was inserted into the ET lumen before the HA injections, and in A5 and A6 after the HA injections. The results of the IVUS method for ET imaging were published by Oppel et al. [38].

### 3.1. Endoscopy

The endoscopic evaluation of the TMs and the pharyngeal ET entrances was successfully performed for all eight animals during the GAs.

#### 3.1.1. TMs

In four cases, the full vision of the TM was not achieved (Table 2). In general, not all TMs could be fully assessed and only the visible part of the TM was analyzed. This study showed that normal and abnormal findings could occur on the TMs of the stent and the control side at all observation times. During the 2nd GA prior to the HA injection, normal TMs were found in seven out of eight cases on the control side and six out of eight cases on the later stented side. The 3rd GA showed five out of eight normal TMs on the control ears and four normal out of seven assessable TMs prior to the stent insertion. The half-time check revealed five out of eight normal TMs on the control side and four out of eight normal TMs on the stent side. During the last follow-up, four out of eight TMs appeared normal for the control side and two out of six on the stent side. Animal A7 showed fluid accumulation in front of the TM in the 3rd GA on the control side. In subsequent controls, this side was rated to be moderately scarred (S) and distinctively scarred (dS).

#### 3.1.2. HA Depot

The positioning of the HA depot was successful in 15/16 ET sides. The left side of animal A2 could not be injected with HA due to complicated vision and the individual anatomy of the sheep’s nasal and pharyngeal cavity. Only 1.47 mL of HA could be injected on the control side of A1. Considering all ET injections (n = 15, without the left side of A2), an average of 2.86 ± 0.72 mL HA was injected on each side, of which 2.99 ± 0.69 mL was injected on the stent side and 2.70 ± 0.78 mL was injected on the control side (Table 3).

#### 3.1.3. Stent Insertion Process

**Figure 2:**
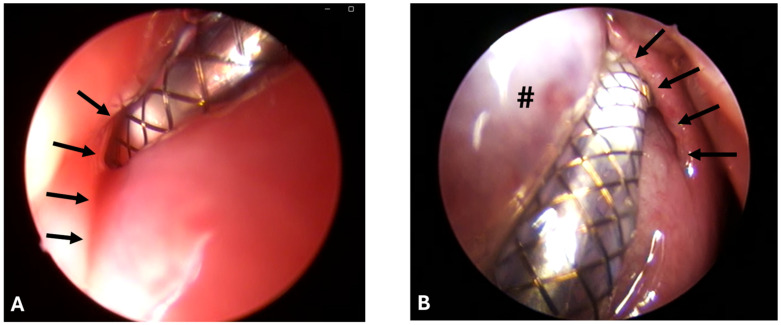
Pharyngeal ET orifices with stent tool inserted during stent placement. Arrows indicate the ET opening. (**A**) Animal A6 on the left side with swollen mucosa and bad vision. (**B**) Animal A8 during stent insertion on the right side with protruding mucosa/HA depot (#) and good endoscopic vision.

Stenting was successful in eight out of eight cases and conducted always by the same surgeon. However, the ET was difficult to access due to the accumulation of clear secretions, the associated poor visibility, and protruding mucosa (Figure 2A,B).

For animal A8, a second stenting approach was needed as the stent was pulled out of the ET while removing the application tool after the first approach. This resulted in a change of ET side because of low visibility due to small mucosal bleeding. In animals A2 and A7, stent release and, in A7, removal of the application tool from the ET lumen were difficult. The desired insertion depth (100%) was reached for animals A4, A5, A6, and A7; nearly reached (>50%) for animals A1, A3, and A8; and not evaluable for A2 due to the secretion and bulging HA depot. The average tool angle used was 30.63° (Table 4).

#### 3.1.4. Opening Grade of ET

**Figure 3:**
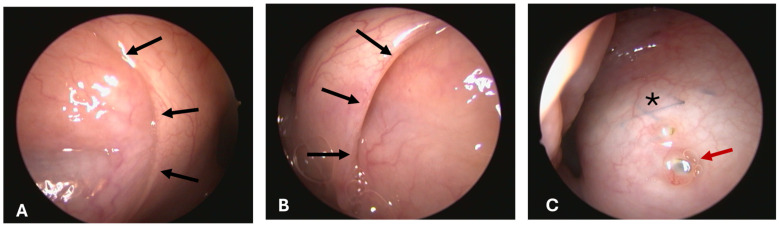
Endoscopic findings of pharyngeal ET openings at the 5th GA. Black arrows indicate the ET opening. (**A**) Animal A5 with normal secretion and closed ET opening on the control side. (**B**) Animal A5 with slightly open stent side. (**C**) Animal A3 with visible stent (asterisk) and perforation of the X-ray marker (red arrow) through the mucosa.

All pharyngeal ET entrances (16/16) were physiologically closed before the HA injection and before the ET stent was inserted into the lumen on stent sides (1st, 2nd, and 3rd GA) (Figure 3A). Only the stented ETs of 2 animals appeared slightly open after the procedure at isolated time points (Table 5). The ET entrance of A6 was slightly open directly after the stent insertion (3rd GA). For animal A5, the entrance was also slightly open in the last follow-up at 3 months after stent insertion (5th GA) (Figure 3B). No ET openings were found to be distinctively or severely open. Animal A3 had stent struts visible through the mucosa in the 5th GA barely perforating the mucosa (Figure 3C).

#### 3.1.5. Secretion

It was difficult to distinguish between secretion from the ET lumen and the general secretion of the nasopharyngeal cavity. The bulging mucosa of the HA depot area trapped more secretions in the nasopharynx. Overall, animals showed clear, serous secretion in the nasopharyngeal area (no side allocation possible) throughout the entire experiment. However, an attempt to categorize ET secretion was made (Table 6).

**Figure 4:**
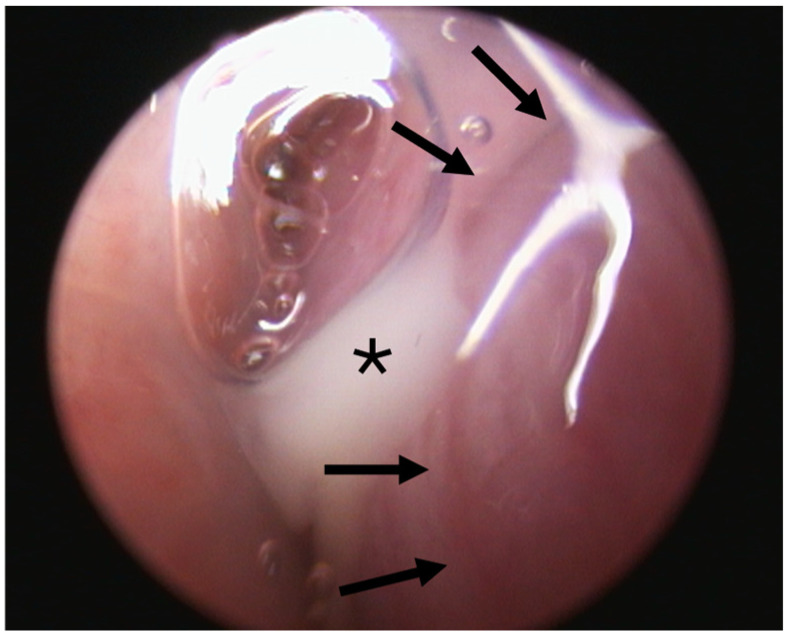
ET opening (left side) of animal A6 during the 3rd GA prior to stent insertion with abnormal secretion (asterisk) associated with the ET lumen. Arrows indicate the ET opening.

Two out of eight animals showed abnormal secretion associated with the ET lumen. Animal A6 had abnormal secretion in the 3rd GA (Figure 4). Prior to stent insertion, there was an outflow of opaque, mucous secretion visible on the side that was later stented in this GA. At the follow-ups (4th and 5th GAs), secretions appeared normal on both sides. Animal A8 showed abnormal secretion on the stented side from the half-time follow-up until the end of the study. All other animals showed only clear, serous secretion during the check-ups of the pharyngeal openings.

For animal A5, a defect of the mucosa with accumulating mucous secretion was detected rostral to the ET entrance in the 3rd GA prior to the stent insertion. The defect matched the location of the previous HA injection site. For the following GAs, no defect and no mucous adhering to the mucosa could be detected.

### 3.2. Tympanometry

The measurements of middle ear ventilation by tympanometry and, thus, indirectly the function of the ET worked reliably throughout the entire study period. Before the application of HA, all middle ears (16/16) appeared regularly ventilated.

#### 3.2.1. Prior to the Stent Insertion/after HA Injection

The tympanometry measurements after the HA injection showed that not all middle ears had impaired middle ear ventilation; i.e., not all ETs had a triggered ETD before the 3rd GA. This affected the selection of the stent and control sides. For the future stent sides, all animals had a possible ETD prior to the stent insertion (week 0). Seven out of eight animals showed type B tympanograms, and one animal (A3) had a type C tympanogram. On the control sides, four out of eight animals had type B tympanograms, one out of eight had a type C tympanogram, and three animals had no confirmed ventilatory dysfunction and showed physiological type A tympanograms. Sheep A2 did not receive HA injections on the control side but showed ventilation dysfunction up to week 2. This means that the tympanometry measurements for the control sides only indicated a possible ETD due to the injection of HA for four out of eight animals.

#### 3.2.2. After Stent Insertion till End of Study

The ventilation status of the middle ear changed over time (Table 7). Three of the four animals with bilateral ETD (excluding A2) showed re-ventilation of the middle ear first on the stent side compared with the control side (physiological type A of the stent side: A3: week 8, A4: week 5, A7: week 6). Animals A5 and A6 had even earlier ventilation at 3 and 4 weeks after stent placement, but no blocked controls were available for comparison. On the stent side, three animals (A1, A2, A8) showed type B tympanograms until week 12. These animals did not reach physiological middle ear pressure during the observation period. For these animals, the control sides showed physiological type A tympanograms at the end of the experiment (including A1 with <2 mL HA and A2 without HA depot). In addition, of the four controls initially measured with ETD after HA injection, two were still abnormal at the end of the observation period (A3, A7). The measured ear canal volume indicated in no case a perforation of the TM.

### 3.3. CBCT

**Figure 5:**
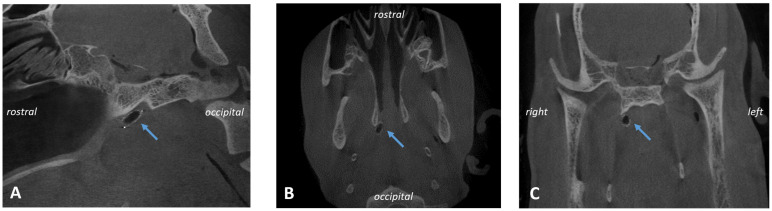
CBCT of animal A1. Ventilated stent in longitudinal (**A**,**B**) and cross section (**C**) of the sheep skull indicated by blue arrows.

CBCT analysis (Figure 5) showed that seven out of eight stents were found located in the cartilaginous part of the ET and, therefore, did not dislocate during the 3-month observation period. However, the stent of A7 was not placed at the desired location. It did not direct up to the middle ear as it had a more downward (distal) twist. All stents were nearly deployed to their intended tubular shape (Table 8). No stent fractures occurred.

In addition, the middle ear ventilation status was assessed. For the controls, six out of eight middle ears were free and air-filled, whereas A3 and A7 presented as not air-filled. The stented sides had three out of eight free middle ears, three partially filled middle ears, and two filled middle ears (for individual results, see Section 3.5). Even though the stent of A7 was not placed correctly, it had a free middle ear on the stent side.

### 3.4. Histology

Histological analysis of the sections was possible for almost all defined sections. However, no S0 sections were available for animals A2 and A4 because the stent was placed too close to the entrance of the ET. Animal A2 had no HA depot on the control side, so these sections were not available. Animal A7 was excluded from histological analysis because of the incorrect placement. Due to the stent being in the surrounding tissue instead of the ET lumen, the stent was fully ingrown with tissue in section S2. However, the ET showed similar residual lumina on both sides with L_T_ = 1.05 mm^2^ and L_C_ = 1.11 mm^2^. The epithelial score of section S2 for the quadrants Q1–Q4 was 4 as the stent was fully ingrown due to the incorrect placement. The control side to section S2 scored 1.

#### 3.4.1. HA Depot

**Figure 6:**
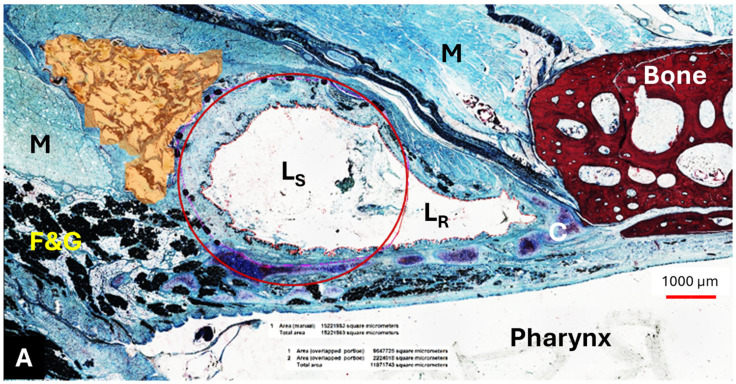

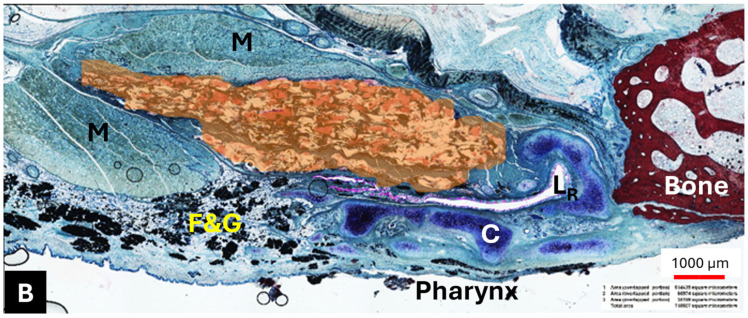
The location of the HA depot (colored in orange) in animal A4 on the stent ((**A**) mirrored view) and control side (**B**) for section S2. M—muscle; F&G—fat and glandular tissue; C—ET cartilage; L_S_—stent lumen; L_R_—residual ET lumen; red circle—approximating the stent struts. The black dots in the vicinity of the circle are the stent struts.

The HA appeared as an amorphous substance interspersed with loose connective tissue. It was located predominantly lateral to the lumen of the ET (Figure 6A,B) and extended like a strand along the length of the cartilaginous ET. The mean HA area (A_HA_) of the sections decreased from S_HA_ to S4 (Table 9).

**Figure 7:**
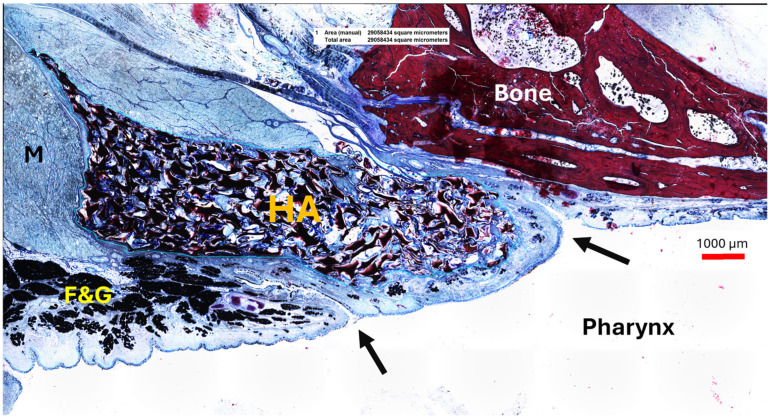
A2 with HA depot in cutting section S_HA_ on the stented side lying in the ET opening augmenting the mucosa. The arrows indicate the beginning of the ET opening and its crescent shape. HA—hyaluronic acid interspaced with connective tissue; M—muscle; F&G—fat and glandular tissue.

The HA area in the S_HA_ cutting section did not differ from its control (*p* > 0.9999); this also applied to S2 and its control, which were also analyzed using Wilcoxon’s matched pairs signed rank test (*p* = 0.3125). The majority of the HA area was found in section S_HA_, located at or in front of the ET entrance and thus anterior to the stent (Table 9, Figure 7). However, the statistical analysis using paired *t*-test and Wilcoxon’s matched pairs signed rank test showed that the HA depot in section S_HA_ is only significantly larger than in sections S3 (*p* = 0.0313) and S4 (*t*(5) = 2.869, *p* = 0.0350). The other sections showed no significant differences to section S_HA_ (S0: *p* = 0.3125; S1: *t*(5) = 2.313, *p* = 0.0687; S2: *t*(5) = 2.536, *p* = 0.0521).

#### 3.4.2. Lumen

The mean value of the ET lumen (sections S0, S4) and the total ET lumen (sections S1, S2, S3) increased from section S0, in front of the stent, to section S2 in the center of the stent, and then decreased to the smallest lumen in section S4 behind the stent (Table 10).

Mixed effects analysis identified significant lumen differences between the histological cutting sections (*F*(0.9884, 5.535) = 21.83, *p* = 0.0043). Sections S1 and S2 had a significantly larger ET lumen than the control (S1: *p* = 0.0437; S2: *p* = 0.0075). The other sections did not differ from the control (S0: *p* = 0.1787; S3: *p* = 0.1883, S4: *p* = 0.7456). Moreover, section S2, the center of the stent, had a significantly larger total lumen area than sections S0 (*p* = 0.0240), S3 (*p* = 0.0061), and S4 (*p* = 0.0015). In addition, section S1 was significantly larger than S4 (*p* = 0.0181). The remaining cutting sections were not different from each other (S0 to S1 (*p* = 0.1709), S3 (*p* = 0.3236), and S4 (*p* = 0.0897); S1 to S2 (*p* = 0.1715) and S3 (*p* = 0.0729); S3 to S4 (*p* = 0.4901)).

The lumen of the stent was in all animals and on both sides lined with epithelium. The struts were covered with epithelium too. The epithelial score of the quadrants was neither statistically different between each other nor from the control side (*p* = 0.1481; Friedman test). Only quadrant 4 showed a non-significant increase in the score compared with the other quadrants and the control (Table 11).

#### 3.4.3. Tissue and Secretion

Tissue ingrowth into the stent occurred in all stent sections. The areas of ingrown tissue for cutting sections S1–S3 (supported by the stent) were 9.81 ± 4.42 mm^2^ (S1), 7.42 ± 3.20 mm^2^ (S2), and 4.87 ± 1.38 mm^2^ (S3). Therefore, the mean tissue area decreased from section S1 up to section S3 at the tapered stent end. The amount of secretion in section S2 covers 0.61 ± 0.54 mm^2^. The corresponding controls contain 0.10 ± 0.05 mm^2^. The paired *t*-test showed no difference between the secretions of the stented and the control side (*t*(6) = 2.445, *p* = 0.0501).

### 3.5. Further Observations

Animals with type A tympanogram also showed free or partially filled MEs in CBCT (Table 12). All filled MEs for the control (two out of eight) and the stent side (two out of eight) in CBCT had also ventilation disorders shown in the tympanogram (type B). Ears (three out of eight) with partially filled MEs on the stent side had two type A and one type B tympanogram.

Tissue ingrowth occurred in all animals for sections S1–S3. However, all animals, including persisting type B tympanograms, had a remaining ET Lumen (L_T_) in the histology evaluation. In addition, the measured total lumen (L_T_) of each animal was always higher than in the corresponding control section (L_C_) with the exception of A7. Animal A7 had the lowest L_T_ measured, and CBCT showed that the stent was placed incorrectly and mostly ingrown in the surrounding tissue. The lumen, therefore, represents the remaining ET lumen, which levels with the corresponding control section.

The control side of animal A8 showed no abnormal values after undergoing a first stent approach ending in pulling the stent out of the lumen during the tool removal. It ended with a physiological tympanogram, a free middle ear, and normal amounts of secretion. In this ear, the middle ear was ventilated again starting in week 3 after stent insertion.

Furthermore, the highest mean HA area (A_HA_) was measured for section S_HA_, which is found anterior of stent sections S1–S3 (compare Figure 1), including section S2 with the highest mean lumen (L_T_) of the stented ET (compare chapter 3.4.). Animal A1 had the lowest HA injection volume (1.47 mL) on the control side, and this is accompanied by a free middle ear. This animal also showed type A tympanograms after the HA injection for the control side.

**Figure 8:**
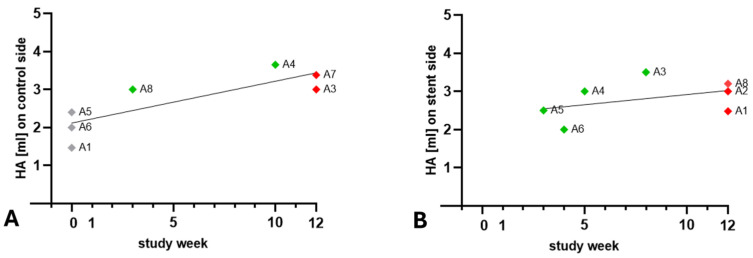
Injected HA volume and weeks needed to restore physiological middle ear pressure (type A tympanogram) for the control (**A**) and the stent side (**B**). Included are animals with regained type A tympanograms (green) and animals with type B tympanogram at the end of the observation period (red). A2 is not displayed in part (**A**) as it had no HA injection for its control. A7 is not displayed in part (**B**) due to the incorrect stent placement. The control sides of animals A1, A5, and A6 had no measurable ETD after the HA injection (grey).

Pearson correlation indicated that the HA injection volume and the time needed to regain physiological type A tympanogram are correlated for the control side (*r* = 0.8173; *p* = 0.0248). Animals A1, A6, and A5 with lower HA volumes already had type A tympanogram in week 0, whereas animals A8 and A4 had measured type A tympanograms in weeks 3 and 10 (Figure 8A). On the stent side, no correlation (*r* = 0.4220; *p* = 0.3457) between the injected volume and the week of re-ventilation of the middle ear was found (Figure 8B).

## 4. Discussion

The aim of this study was to investigate stent placement in a mechanically blocked ET and to gain further insight into the function of a tapered ET stent designed for the human ET.

During the study, the animals showed no clinical abnormalities that could be attributed to the HA injection or the stent. No tilting of the head or any other behavioral abnormalities were observed. The only animal with an abnormal score probably suffered from an acute lower respiratory infection, which suddenly became apparent with fever and an elevated respiratory rate. As the animal responded effectively to antibiotic treatment, it is most likely that the cause was infectious and not experimental. However, influences of the previous anesthesia, such as aspiration of saliva during the waking phase or the stent procedure itself, cannot be completely excluded as a cause of infection.

A method described earlier [29] was applied to both ET sides to create a mechanically induced ventilation disorder, and subsequently, the stents were inserted unilaterally into the ET lumen via a transnasal approach. However, animal A2 could only be injected with HA on one side. This was due to anatomical abnormalities of the nasal cavity, particularly the nasopharynx. The mucosa appeared swollen, and the nasopharynx narrowed compared with the other sheep in the study. The exact reason for this can only be surmised, but allergic or dust-induced rhinitis could be a reason for the changes. The animal was otherwise clinically unremarkable. This finding is also supported by the ventilatory dysfunction of the middle ear measured on the control side up to week 2. However, the contralateral mucosal swelling after the HA injection could have also added to the ETD on the control side.

All stents could be successfully inserted into the ET lumen. However, the stent insertions were performed under changed conditions. Compared with previous stenting approaches in healthy ETs [24,25,28,39], all stents were inserted into a dysfunctional ET. The injection of the HA produced mucosal swelling at the ET opening, augmented the ET opening, and possibly narrowed the ET lumen. In addition, clear secretion was omnipresent in the nasopharynx during the stent insertion process, probably increasingly retained by the mucosal swelling due to the HA depot. This could have also influenced the stent insertion process in terms of reduced vision and different insertion angles. Compared with a recent study [28] of stenting physiological sheep ETs with an average insertion angle of 24.6° (n = 24), the one of the current study was determined to be a little higher with 30.63° (n = 8). It is possible that a larger angle was required to allow the stent tool to follow the ET lumen despite the blockage near the entrance and alongside the ET lumen. In addition, one stent was inserted into the ET lumen but placed into the surrounding tissue. It is assumed that the insertion process was not carried out as planned, and the tool was accidentally pushed forward during the release process. However, this could not be proven due to the reduced vision in the video endoscopy. This incorrect insertion could also have been influenced by poor vision due to the above-mentioned swelling and secretion. Moreover, stent insertion did not induce patulous ETs. Most of the ETs appeared closed, and only one animal showed a slightly open ET at the final follow-up such that, in this animal, a patulous ET after 3 months of implantation cannot be excluded. Not inducing patulous ETs was desired as these could lead to autophony or otitis media due to ascending pathogens and therefore should be prevented when stenting human ETs [40,41]. In one animal (A3), stent struts were visible through the mucosa after 3 months (compare Figure 3C). Insertion depth was not fully reached in this animal (compare Section 3.1.3). This means that the stent was positioned closer to the pharyngeal orifice than intended. A smaller head/shorter ETs is the typical reason for this [28]. We did not find any indication regarding difficulties during the removal of the insertion tool in either the surgical reports or the endoscopic documentation of the insertion. Therefore, most likely, the position of the stent too close to the pharyngeal orifice is the reason for the submucosal appearance of the stent. Sheep do not have a torus tubarius. At this place, there is only a very thin layer of tissue between the ET and the pharynx.

The IVUS procedure performed in three animals, which was part of another experiment, most likely did not influence the final histological results. The same applies to insertion trauma that may have occurred during stent implantation except generating a via falsa. The reason for this assumption is that the mucosa regenerates very quickly. A recent study [42] showed that the initial insertion trauma to the ET, e.g., decrease in lumen and epithelial hyperplasia, induced by BET was fully recovered at 12 weeks after BET. This is consistent with the results of the epithelial analysis of all quadrants from the stented side, which showed no significant deviation from the control (see Section 3.4.2). The examination showed good epithelization of the stent struts in all quadrants, which is necessary for the ET drainage function through the cilia of the pseudostratified columnar epithelium [4,5]. These findings also apply to the initial stent insertion approach of animal A8. The stent was pulled out of the ET lumen during the tool removal of the ET, and the subsequent hemorrhage and decline of endoscopic vision forced a change of the stent side. The mucosal hemorrhage and possible trauma to the mucosa due to the withdrawn stent is also assumed to have had no influence on the histological results at the end of the 3-month observation period due to the reasons mentioned above. Additionally, results showed no abnormal values for the control side of A8, neither for the ET lumen nor for the CBCT evaluation and the final tympanogram.

After stenting blocked ETs, there were only few abnormal discharges associated with the ET lumen other than the physiological clear secretions present in the nasopharyngeal cavity. One animal (A6) showed abnormal secretion prior to stent insertion (3rd GA), which was absent in subsequent controls. It seems plausible that the then-inserted stent helped drain the middle ear. This animal had type B tympanograms on the stent side until week 3 and physiological middle ear pressure with type A tympanogram afterwards. It also had free middle ears for the control and the stent side on the final day. Unfortunately, ventilation disruption could not be induced on the other side. Therefore, a direct comparison between the control and the stent side regarding middle ear ventilation was not possible. The other animal with abnormal secretion was A8, who had opaque, mucous discharge in the half-time and final follow-up on the stented side. This animal also showed a filled middle ear and a type B tympanogram on the stented side at the end of the study. Apart from this, all other endoscopic controls showed normal secretion. This was also confirmed by the histological results, as only small areas taken by secretion were measured in the ET lumen. However, histological analysis measured ingrown tissue in all three stent sections (S1–S3). Although cutting section S3 had the smallest amount of granulation tissue, it must be considered that the stent cross section here is also the smallest due to the tapered end of the stent. Tissue ingrowth is a common finding when stents are histologically analyzed. For example, studies on tracheal stents in dogs [43] and ET stents in a porcine model [39] or rat urethras [44] showed ingrowth of tissue into the stent lumen. Even if granulation tissue occurs in stents, it should be kept to a minimum to avoid creating an obstruction in the worst case. Some possible approaches to reduce tissue formation in stents by the application of substances, such as EW-7197 [45] or Sirolimus [39], showed promising first results and should be taken into account for the ET stent in future studies.

In addition to tissue growth, the lumen of the ET also follows the shape of the stent. The ET narrows to the smallest part of the lumen [2], the isthmus. Therefore, the stent shape adapts to the anatomy and, consequently, also the lumen in the stent area. Despite the existing HA blockage, the histological results prove that the lumen was increased in the stent sections compared with the control. Thus, the lumen analysis showed that ventilation through the ET should be possible for all animals. Unfortunately, this was not always the case. The distribution of the HA is presumably the reason for that. Detecting HA in the tissue after the observation period was to be expected, as degradation with noticeable volume loss is described for stabilized HA after 6–12 months [30,31]. However, histological analyses showed that the majority of HA was measured in the section (S_HA_) located anterior of the stent and thus close to the pharyngeal ET opening. The HA, located in the lateral wall of the ET, then decreased over the sections until the last section S4 towards the middle ear. This means that, on average, the stent (located in sections S1–S3) was positioned behind the site of the largest accumulation of HA. It is very likely that the amounts of HA located in the sections S_HA_ and S0 contributed to the remaining ETD, as the stent did not cover these areas and therefore could not help to overcome the blockage. A theoretical solution for this experimental setup would be to place the stents farther rostral in the entrance of the ET. However, this would inevitably increase the risk of inducing patulous ETs.

It should also be noted that the CBCT results of the control sides reflect the ETD from study week 12. The two animals with filled control MEs at the end of the study had type B tympanograms persisting from week 0 until week 12. Therefore, ongoing ventilation disorders have probably contributed to mucus accumulation and thus filled MEs and type B tympanograms. However, this relationship appears to be not as clear on the stented side. Here, two out of three MEs with persisting ETD were filled with mucus/tissue, whereas the other ear with ETD (A1) had only a partially filled ME. However, two middle ears of the stented ETs with restored middle ear ventilation were also partially filled. It remains a speculation, but these findings could indicate that mucus was not yet completely drained after restoration, or in the case of the persisting type B tympanogram of animal A1, that restoration of middle ear ventilation would have followed shortly after week 12.

Looking at the tympanometry results, three animals had no confirmed ETD on the control side prior to stent placement. This limited a direct comparison regarding whether the stent side is re-ventilated earlier than the respective control side. In addition, the control side of A2 was not injected, leaving only four animals with bilaterally induced ETD. The results show that five out of eight stented ET sides (A3, A4, A5, A6, A7) had re-ventilation of the middle ear during the observation period despite the HA blockage. However, A7 needs to be excluded as the stent was not found correctly in the ET lumen. Unfortunately, only 2 out of the remaining 4 positive re-ventilations had blocked controls, which could prove an earlier re-ventilation of the stent side than the control side. Only in animal A8, the stented ET was blocked longer than the control with induced ETD. However, this animal was initially stented on the control side. It remains a speculation, but it cannot be excluded that the short-term implantation during the 3rd GA contributed to the early re-ventilation of the control side in week 3. Therefore, the positive outcome is reduced due to the lack of induced ETD on the control sides. Three animals (A1, A2, A8) had ventilation disorders on the stent side until the end of the study. The possible reasons for that are already discussed with the HA depot being distributed with its majority in front of the stent supporting the lumen. It is therefore possible that the full function of the stents could be shadowed in tympanometry data by type B tympanograms induced by the HA blockage directly at the pharyngeal orifice of the ET in front of the stent. There also appears to be a correlation between the amount of injected HA and the time until re-ventilation on the control side. This is not the case on the stent side. For animals A3 and A4, it seems plausible that the stent caused the effect, as in both cases, the control sides were ventilated later. For animals A5 and A6, which were also re-ventilated, this cannot be assessed as the control sides were not blocked. However, there is no clear explanation as to why not all animals showed re-ventilation on the stent side.

## 5. Conclusions

For the first time, stents were successfully placed in an ET augmented with HA to cause ETD. The results showed early encouraging evidence of the stent function. Despite the induced ETD, the stents reached almost their original shape and did not produce patulous ETs. Four out of seven correctly stented animals with ETD showed re-ventilation of the middle ear after stent placement. However, compared with the control side, there were only two cases of earlier re-ventilation, as not all controls had pre-existing ETD. Moreover, the stent enlarged the ET lumen compared with the control. Unfortunately, this study also showed that the model of stent placement and HA injection did not necessarily fit. The HA was mainly localized in the area anterior of the stent, and the stent was therefore unable to perform its full function over the entire length of the blockage. To examine the stent function in further studies, the stent positioning should be individually adjusted to the blockage and/or even placed close to or in the ET opening, if necessary. Consequently, this study provides encouraging first results of inserting stents in a model of mechanically induced ETD.

## Figures and Tables

**Table 1:** Overview of the consecutive study steps including 5 endoscopic procedures under GA.

Study Step		Study Proceedings
Start of study	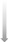	Veterinary check, quarantine, handling, and training
1st GA *		Initial cleaning of EACs, inspection of TMs and ET entrances (study week −3)
2nd GA		Cleaning ^1^ of EACs, inspection of TMs and ET entrances, *bilateral HA injection* (study week −1)
3rd GA		Cleaning ^1^ of EACs, inspection of TMs and ET entrances, *unilateral stent insertion* (study week 0)
4th GA		Cleaning ^1^ of EACs, inspection of TMs and ET entrances (1.5 months after stent insertion)
5th GA		Cleaning ^1^ of EACs, inspection of TMs and ET entrances (3 months after stent insertion), euthanasia in GA
Post-mortem		CBCT, sampling, histology

*—start of weekly tympanometry on both ears after initial cleaning of EACs until 5th GA; ^1^—if needed; TM—tympanic membrane.

**Table 2:** The condition of the tympanic membranes at each stage of the study for the control and stent sides.

TM	2nd GA: Prior HA		3rd GA: Prior Stent		4th GA: Half-Time		5th GA: Final	
Animal	Control	Stent	Control	Stent	Control	Stent	Control	Stent
A1	~	B	~	B	~	B	B	B
A2 *	~	~	~	~	~	~	~	~
A3	~	~	S, B	S, B	S	S	mS	dS
A4	~	mS ^#^	~	~	mS	mS	mS	mS
A5	~	~	~	n.a.	~	~	~	~
A6	~	~	~	~	~	~	~	n.a.
A7	mS	n.a.	F	mS, B	S, B	mS	dS	mS
A8	~	~	S, B	~	~	~	~	n.a.

TM—tympanic membrane; *—HA only on stent side; ~—TM partly visible and normal; F—fluid, possible rupture of TM; mS—mild scarring; S—moderate scarring of TM; dS—distinct scarring; n.a.—not assessable; B—bulging of TM; #—minor dot-shaped hemorrhage.

**Table 3:** Injected hyaluronic acid volumes for each animal.

Animal ID	HA Control	HA Stent Side
A1	1.47	2.48
A2	0 *	3.0
A3	3	3.5
A4	3.65	3
A5	2.4	2.5
A6	2.0	2.0
A7	3.38	4.25
A8	3.0	3.2

Hyaluronic acid [mL] per ET side for each animal (n = 8); *—HA injection was not possible; underlined—ETD present prior stent insertion.

**Table 4:** Angles of application tool tip used for stent insertion.

**Animal**	**A1**	**A2**	**A3**	**A4**	**A5**	**A6**	**A7**	**A8**
**Tool angle**	35°	20°	40°	30°	30°	30°	40°	20°

**Table 5:** Opening grades of the pharyngeal ET entrance after the stent insertion.

Opening	3rd GA: After SI		4th GA: Half-Time		5th GA: Final	
Animal	Control	Stent	Control	Stent	Control	Stent
A1	closed	closed	closed	closed	closed	closed
A2	closed	closed	closed	closed	closed	closed
A3	closed	closed	closed	closed	closed	closed ^#^
A4	closed	closed	closed	closed	closed	closed
A5	closed	closed	closed	closed	closed	open ^S^
A6	closed	open ^S^	closed	closed	closed	closed
A7	closed	closed	closed	closed	closed	closed
A8	closed	closed	closed	closed	closed	closed

SI—stent insertion; ^#^—stent strut visible through and X-ray marker perforating the mucosa; ^S^—slightly.

**Table 6:** Secretion at the pharyngeal Eustachian tube entrance.

Secretion	2nd GA: Prior HA		3rd GA: SI		4th GA: Half-Time		5th GA: Study End	
Animal	Control	Stent	Control	Stent	Control	Stent	Control	Stent
A1	n	n	n	n	n	n	n	n
A2	n	n	n	n	n	n	n	n
A3	n	n	n	n	n	n	n	n
A4	n	n	n	n	n	n	n	n
A5	n	n	n	n ^1^	n	n	n	n
A6	n	n	n	a	n	n	n	n
A7	n	n	n	n	n	n	n	n
A8	n	n	n ^2^	n	n	a	n	a

SI: stent insertion; secretion score: n—normal (clear, serous); a—abnormal secretion (opaque, mucous) associated with ET lumen; ^1^—mucous/pus adhesion to perforated mucosa located anterior of the ET opening at the former HA injection site; ^2^—small bleeding after first stent insertion approach (stent was pulled out of the ET, change of stent side).

**Table 7:** Tympanogram types for the stent and control sides throughout the study.

			Study Week											
		0	1	2	3	4	5	6	7	8	9	10	11	12
**HA + Stent**	A1	B	B	B	B	B	B	B	B	B	B	B	B	B
	A2	B	B	B	B	B	B	B	B	B	B	B	B	B
	A3 ^RV^	C	B	B	B	B	B	B	B	A	A	A	A	A
	A4 ^RV^	B	B	B	B	B	A	A	A	A	C	A	A	A
	A5	B	B	B	A	A	A	A	A	A	A	A	A	A
	A6	B	B	B	B	A	A	A	A	A	B	B	A	A
	A7 ^RV^	B	B	B	B	B	C	A	C	A	A	C	A	A
	A8	B	B	B	B	B	B	B	B	B	B	B	B	B
	**HA**		**HA + Stent**											
**HA Control**	A1 *	A	A	A	A	A	A	A	A	A	B	B	A	A
	A2 ^#^	B	B	C	A	A	A	A	A	C	A	A	A	A
	A3	C	C	B	B	B	B	B	B	B	B	B	B	B
	A4	B	B	B	B	B	B	B	B	B	C	A	A	A
	A5 *	A	A	A	A	A	A	A	A	A	A	A	A	A
	A6 *	A	A	A	A	A	A	A	A	A	A	A	A	A
	A7	B	B	B	B	B	B	B	B	B	B	B	B	B
	A8	B	B	B	A	A	A	A	A	A	A	A	C	A
	**HA**		**HA**											

Week 0: with HA prior to stent insertion; week 1: 1st measurement with stent; week 6: half-time check (4th GA); week 12: final check (5th GA); ^RV^—re-ventilation at first on the stent sides compared with controls with ETD; *—control sides without measurable ETD in tympanometry with injected HA; ^#^—no HA injection; green—physiological type A tympanogram; yellow—abnormal type C tympanogram; red—abnormal type B tympanogram.

**Table 8:** CBCT measurements of stent cross-sections [mm] and length [mm].

	Mean	SD
length	13.6	0.1
cs1 max.	5.1	0.4
cs1 orth.	4.6	0.5
cs2 max.	5.2	0.2
cs2 orth.	4.8	0.2
cs3 max.	3.2	0.1
cs3 orth.	2.9	0.3

cs—cross section; max.—maximal; orth.—orthogonal.

**Table 9:** Area of hyaluronic acid [mm^2^] of animals A1–A8 of the histological sections.

Section	S_HA_	S0	S1	S2	S3	S4
Stent side	10.70 ± 10.43	8.43 ± 8.63	6.06 ± 5.46	4.34 ± 4.25	1.05 ± 1.01	0.69 ± 0.94
Control side	12.81 ± 13.01			5.39 ± 4.25		

Data presented as mean ± SD without A7 for the stent sections and A2 for the control side. S1, S2, and S3 cover the stent.

**Table 10:** Area [mm^2^] of the lumen (L) and total ET lumen (L_T_) of the histological sections.

Section	S0	S1	S2	S3	S4
Stent side	7.11 ± 4.71	8.76 ± 4.98	10.89 ± 4.16	3.05 ± 1.11	2.18 ± 1.65
Control side			1.46 ± 1.0		

Data are shown as mean ± SD. Without A7 due to incorrect stent placement. Individual values for S2 (stent and control) are provided in Section 3.5.

**Table 11:** Epithelial score of the applied quadrants.

Quadrant	Score
Q1	1.3 ± 0.5
Q2	1.3 ± 0.5
Q3	1.1 ± 0.4
Q4	1.6 ± 0.8
control	1.1 ± 0.4

Data presented as mean ± SD, excluding A7.

**Table 12:** Side-by-side results of the last follow-up measurements.

	Week 12 Tympanogram		Week 12 CBCT Middle Ear Status		Week 12 Secretion in 5th GA		L_T_ [mm^2^] Section S2	L_C_ [mm^2^] Section S2
Animal	Stent	Control	Stent	Control	Stent	Control	Stent	Control
**A1**	B	A	partially filled	free	normal	normal	11.33	0.96
**A2 ^1^**	B	A	filled	free	normal	normal	8.15	0.36
**A3**	A	B	partially filled	filled	normal ^3^	normal	6.19	1.78
**A4**	A	A	partially filled	free	normal	normal	14.97	0.65
**A5**	A	A	free	free	normal ^4^	normal	17.44	2.86
**A6**	A	A	free *	free	normal	normal	7.06	2.69
**A7 ^2^**	A	B	free	filled	normal	normal	1.05	1.11
**A8 ^5^**	B	A	filled	free	abnormal	normal	11.11	0.92

*—thickened ventral osseous bulla wall; ^1^—unilateral HA depot (stent side); ^2^—incorrect stent placement; ^3^—mucosa-perforating stent in 5th GA; ^4^—slightly open ET in 5th GA; ^5^—change of stent side after initial stent approach; green—animals with earlier re-ventilation of the middle ear compared with control.

## Data Availability

The original data of this study are available on request from the corresponding author.
